# Development of Support Layers and Their Impact on the Performance of Thin Film Composite Membranes (TFC) for Water Treatment

**DOI:** 10.3390/polym15153290

**Published:** 2023-08-03

**Authors:** Qing Zhang, Rui Zhou, Xue Peng, Nan Li, Zhao Dai

**Affiliations:** 1 School of Chemical Engineering and Technology, Tiangong University, Tianjin 300387, China; 2 State Key Laboratory of Separation Membranes and Membrane Processes, Tianjin 300387, China; 3 School of Chemistry, Tiangong University, Tianjin 300387, China

**Keywords:** substrate, support layer, electrospinning, thin-film composite membrane, phase inversion

## Abstract

Thin-film composite (TFC) membranes have gained significant attention as an appealing membrane technology due to their reversible fouling and potential cost-effectiveness. Previous studies have predominantly focused on improving the selective layers to enhance membrane performance. However, the importance of improving the support layers has been increasingly recognized. Therefore, in this review, preparation methods for the support layer, including the traditional phase inversion method and the electrospinning (ES) method, as well as the construction methods for the support layer with a polyamide (PA) layer, are analyzed. Furthermore, the effect of the support layers on the performance of the TFC membrane is presented. This review aims to encourage the exploration of suitable support membranes to enhance the performance of TFC membranes and extend their future applications.

## 1. Introduction

The development of industrial civilization has been a primary goal for human endeavors, intending to enhance productivity and economic growth. It has become a key impetus for societal progress toward modernization. Moreover, it is noteworthy that challenges manifest in various forms, among which water pollution stands out as one of the most pressing global issues. Nonetheless, it is widely recognized that water holds a pivotal position not only in sustaining life but also in facilitating industrial production. Consequently, it becomes an urgent matter that demands global attention and prompt action [1,2,3]. The discharge of wastewater from textile dyeing, along with the presence of heavy metals, chemical fertilizers, and pesticides, is widely recognized as the main source of water pollution. Therefore, a wide range of methods have been extensively used to enhance water purification through diverse physical, chemical, and biological processes. These methods include adsorption, irradiation, membrane filtration, photocatalytic oxidation, biological degradation, and ion exchange [4]. Among these methods, membrane technology has gained widespread application in desalination to address the emerging problem of water pollution, owing to its high energy efficiency, ease of operation, and low cost [5,6]. 

**Figure 1:**
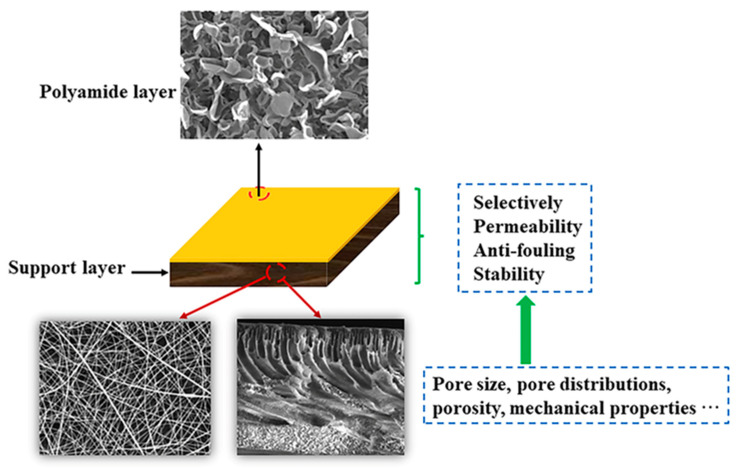
Schematic illustration showing the role of support layers in the TFC membrane.

Traditionally, a dense selective layer and a porous support layer were composed of a thin-film composite (TFC) membrane created using an interfacial polymerization (IP) reaction, which has become a trend due to its rapid, simple, and good separation performance [7,8,9]. The dependence of salt rejection and water permeability in TFC membranes has long been attributed to the selective layer, while the support layer has been recognized as providing mechanical strength, support, and a platform for reactions during operational processes, as illustrated in Figure 1. A TFC membrane can be independently manipulated for the selective and support layers, leveraging their inherent characteristics to maximize its potential [10]. As a result, TFC membrane technology has witnessed rapid development compared to other industrial wastewater treatment membrane technologies [11]. Generally, the commercial market for PA TFC membranes has predominantly revolved around nanofiltration (NF) and reverse osmosis (RO) membranes, particularly with extensive application in the water treatment processes [12,13,14]. In addition, the emergence of forward osmosis (FO) technology, characterized by its lower energy consumption and fouling reversibility, has gained significant attention and widespread use [15,16]. 

The phase inversion method has been regarded as a classical approach to asymmetric membrane fabrication methods since its proposal by Loeb and Sourirajan in the 1960s [17]. Then, the TFC membrane was successfully patented in 1981 by Cadotte [18], which was a remarkable achievement signifying a significant leap forward in the realm of membrane separations, and it has continued to receive the attention of researchers until now [19,20,21]. In this field, the an-fouling, stability, and trade-off between selectivity and permeability in TFC membranes are considered the key current issues [22]. Hence, the majority of research papers have documented techniques to ameliorate these issues by optimizing the selective layer [23,24,25]. For instance, enhancing the hydrophilicity of the PA membrane surface was used to reduce the interaction between the foulant and the selective layer [26,27,28,29]. One limitation of modifying the PA layer is the potential for defects to arise in the selective layer. Consequently, additional approaches are being actively explored by researchers to overcome this limitation. Previously, the substrate membrane was considered to serve as a platform for the formation of the IP reaction and was found to have nearly no influence on the effect of the membrane [15].

Recently, the design of support layers has been considered a more promising strategy, especially in terms of enhancing membrane selectivity and flux, thereby improving their functionality in practical applications. In addition, optimizing the structure and the hydrophilicity of the support layer are effective means to improve the anti-fouling and stability performance of TFC membranes [30,31]. TFC membranes are affected by two main aspects of the substrate membranes, including the selection of the substrate (hydrophilicity/hydrophobicity, mechanical properties, and physical–chemical stability) as well as the structural parameters (types of pores, pore size, pore distributions, porosity, and specific surface area) [18,32,33]. Reducing the thickness of the support layer is beneficial for enhancing water flux but may lead to a reduction in salt rejection [18]. Moreover, the pore structure on the support layer′s surface affects the thickness of the PA skin layer and the possibility of the formation of defects on the PA layer, thereby affecting the separation performance [34,35]. For instance, the effect of the pore sizes ranging from 18 nm to 120 nm in the polysulfone (PSf) support layer on the PA layer′s selective properties were systematically analyzed [36]. The results demonstrated the highest salt rejection of 99% for the PA layer corresponding to the support layer with an 18 nm pore size, whereas the PA layer with the support layer pore size of 120 nm exhibited the lowest salt rejection of 80.5%. A recent investigation conducted by Gao et al. [34] also corroborates this view that the support layer′s pore size affects the selective layer morphologies and overall performance. Specifically, a support layer with a larger pore size of 41.6 nm resulted in a rough PA layer with 250 nm thickness. Conversely, the support layer with a smaller pore size of 7.5 nm produced a smooth PA layer with 180 nm thickness. In addition, an in-depth understanding that the interfacial adhesion between the PSf support layer and the PA layer was achieved using mechanical interlocking. The backflush tests of TFC membranes revealed that mechanical interlocking was not favored by either the support layer with the largest or smallest surface pore size. It was found that when the surface pore size of the support layer lies between the largest and smallest surface pore sizes, it corresponds to better mechanical interlocking to ensure good interfacial adhesion.

This paper reviews the recent advances in traditional porous supports and electrospun nanofiber supports for TFC membranes. It systematically introduces the preparation methods for TFC substrate membranes and summarizes the polymerization, deposition, and adhesion processes of selective layers on the support layers. Moreover, it investigates the effects of support layer properties and modification methods on TFC membrane performance. This review paper aims to highlight the importance of substrate membranes as both essential components and performance enhancers of TFC membranes and to suggest future directions for their optimization and development.

## 2. Fabrication of Supports

The phase inversion method refers to dissolving the polymer in a solvent, forming a homogeneous and viscous casting solution, and then achieving phase separation using a certain physical technology [37]. In recent years, increasing interest has been directed toward electrospun substrate membranes owing to their thickness controllability, high porosity, and open pore structure [38]. It is a process that is based on the interaction between electrostatic force and surface tension. By harnessing the electric field generated with the application of high voltage, a polymer solution is ejected from a needle or nozzle and subsequently solidified, thereby producing fibers [39]. 

### 2.1. Fabrication of Phase Inversion Flat Sheet Supports

**Figure 2:**
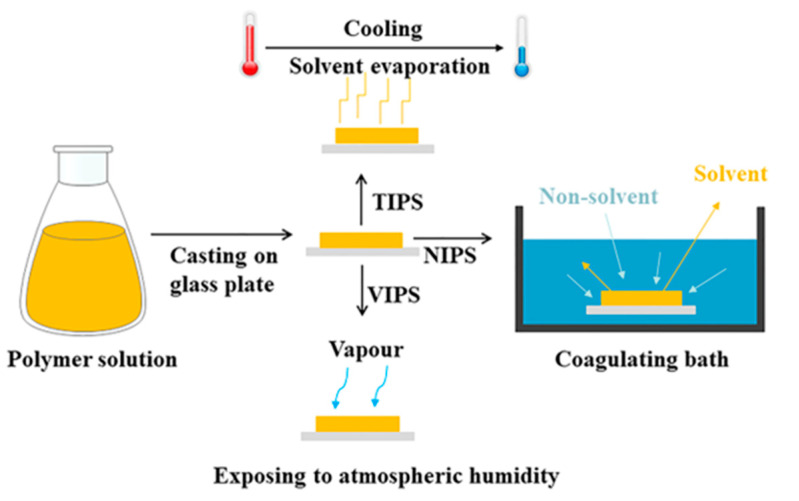
Illustration showing the manufacturing techniques for support layers.

In most cases, porous structures are observed in the finally cured membranes. According to different physical conditions, phase inversion can be divided into non-solvent-induced phase separation (NIPS), also referred to as the immersion precipitation or the Loeb Sourirajan method [40], thermally induced phase separation (TIPS), and vapor-induced phase separation (VIPS). The general mechanism underlying phase inversion is shown in Figure 2. Interestingly, additives play a pivotal role in determining the properties of membranes, as they effectively increase porosity [41] and optimize overall performance [42,43].

NIPS is a process in which a polymer solution is immersed in a non-solvent, leading to the formation of a porous structure [44]. The non-solvent acts on the polymer to precipitate it, resulting in the formation of two distinct phases: a polymer-rich phase and a solvent-rich phase [45]. During the TIPS method, the polymer solution is initially a homogeneous solution at a high temperature but undergoes phase separation when the casting solution is rapidly cooled [46]. The change in temperature alters the solubility of the polymer in the solvent, causing it to separate into polymer-rich and solvent-rich phases [47]. Normally, during the VIPS method, the polymer solution is exposed to atmospheric humidity, where gas non-solvent penetration into the polymer solution causes a phase inversion [48]. In summary, the main difference between NIPS, VIPS, and TIPS lies in the triggering mechanism underlying phase separation.

PSf, polyvinylidene fluoride (PVDF), polyacrylonitrile (PAN), polyethersulfone (PES), polyimide (PI), polyethylene (PE), polypropylene (PP), and cellulose derivatives are commonly used as the substrate membrane polymer for TFC membranes. Moreover, polycarbonate (PC) was used as a substrate for FO TFC membranes by Alihemati et al. [49], which has been reported in only a few studies. This innovative approach demonstrated the potential for expanding the range of possibilities for TFC membrane preparation by exploring less commonly used substrate materials. The aforementioned membrane substrate materials each have their advantages and disadvantages for TFC membrane preparation. For instance, PVDF membranes have high thermal stability and good chemical resistance, but they are hydrophobic, which may result in lower water fluxes [50,51]. Cellulose acetate (CA) is a low-cost hydrophilic polymer membrane, which generally exhibits vulnerability to bacterial and chemical agents [52,53]. However, the negative aspects of these materials can be mitigated or reduced using various modification technologies, such as blending, cross-linking, or surface modification. 

To address these limitations, it is imperative to develop and produce novel substrate membranes with enhanced stability, anti-pollution properties, and separation performance for TFC membranes. The use of multiple conversion techniques should serve as a feasible resolution, whereby the limitations of each phase inversion method can be offset or mitigated by the others. Since the initial use of the non-solvent thermally induced phase separation (N-TIPS) method in 2002 [54], extensive research has been conducted to comprehensively understand this novel technique from various aspects. The N-TIPS method involves first heating the polymer in a diluent at a high temperature, followed by pouring it onto a plate at the same temperature. Subsequently, the polymer solution is immersed in a non-solvent bath with a significantly lower temperature than the polymer’s crystallization temperature [55,56]. Superior membrane performance is achieved using the N-TIPS method, which fabricates membranes with a loose skin layer and a high porosity porous support layer, offering promising prospects for the future [57]. Detailed information regarding the preparation of the traditional substrate membranes can be found in Table 1, which presents a comprehensive overview of the key steps and parameters involved in the fabrication process.

### 2.2. Fabrication of Patterned Electrospun Fiber Supports

**Figure 3:**
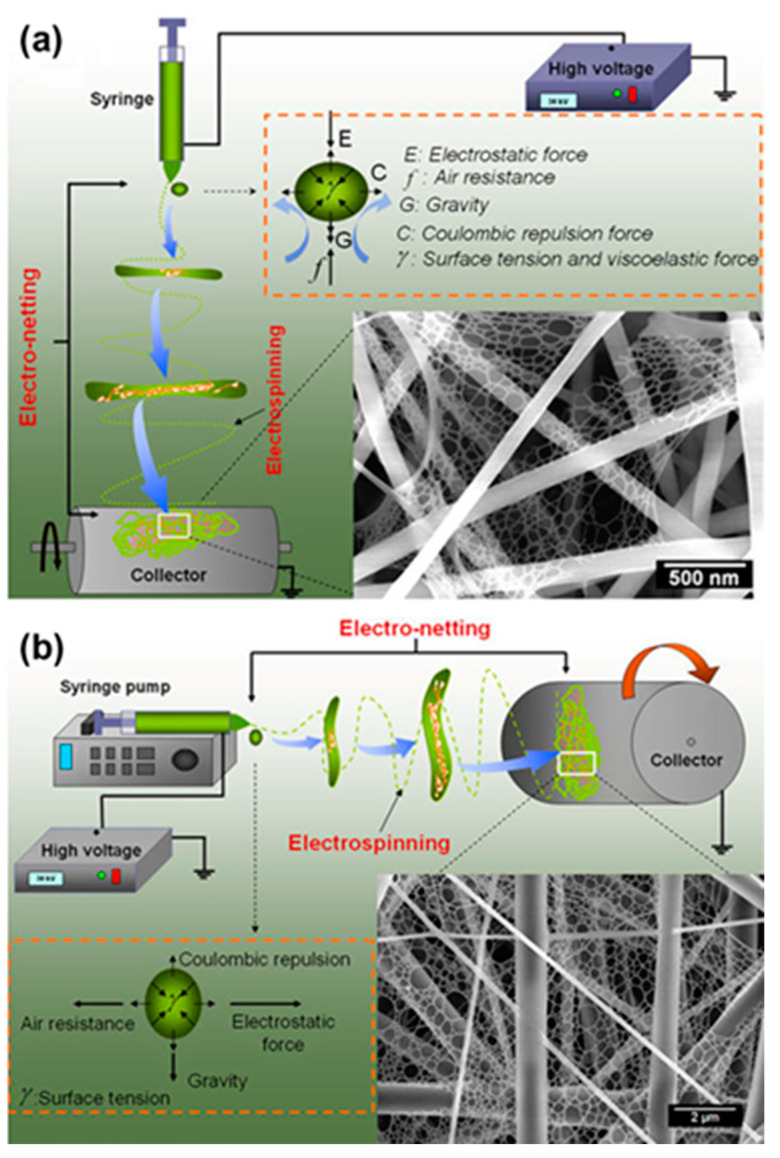
Schematic diagram showing the setup of an electrospinning/netting apparatus: (**a**) typical vertical setup. Reprinted with permission from Ref. [99]. 2011, Wang et al. and (**b**) horizontal setup of an ES apparatus. Reprinted with permission from Ref. [100]. 2011, Yang et al. The insets show drawings of the forces acting on the charged droplet and typical field emission scanning electron microscopy (FE-SEM) images of nanofiber/net (NFN) membranes.

The earliest record of electrospinning (ES) technology was proposed by Formhals in 1934 [89]. However, the breakthrough in understanding and control of ES technology came with Taylor’s contribution [90]. Currently, the ES technique is implemented in two primary setups, namely, the vertical (Figure 3a) and the horizontal (Figure 3b) [91]. In the horizontal setup, the syringe pump is parallel to the ground and produces an electric field vector parallel to the ground is called a horizontal setup, while in the vertical setup, the electric field vector is perpendicular to the ground [92]. The main difference between vertical and horizontal setups is their orientation, which is similar in terms of application. ES technology has been used in a wide range of applications in the textile industry [93], energy and sensor applications [94], antibacterial fields [95], and biotechnology [96]. Generally, the polymer solution in the syringe pump is pushed out at an appropriate speed under the stimulation of a high-voltage power supplier. Simultaneously, nanofibers are formed on the metal collector by solvent volatilization [97]. Both configurations are susceptible to the influence of a diverse range of factors, encompassing not only solution parameters and process parameters but also environmental parameters, alongside other pertinent variables [98]. The intricate interplay of these factors collectively impacts the performance and outcomes of the ES process.

The ES technique, which is used for fabricating nanofiber substrate membranes, exhibits superior characteristics, including a high specific surface area, volume ratio, and open pore structure [101]. The preparation of substrate membranes with these technical advantages can be seen in Table 2. However, it should be noted that the pore size and morphology of electrospun nanofiber membranes can be influenced by preparation conditions and polymer properties. While some smaller or larger pores may be present, a large number of closed pores are usually absent. The production of TFC membranes benefits from the fact that nanofibers are used as an ideal substrate for interfacial polymerization, owing to the aforementioned characteristics.

While ES technology has proven successful in fabricating nanofiber support layers within a laboratory environment, further research and development are imperative to fully capitalize on its potential for large-scale manufacturing. This necessitates the meticulous refinement and optimization of the ES process to enable its ubiquitous commercial application in large-scale manufacturing. A discernible enhancement in the overall performance and properties of TFC FO membranes is brought about by the incorporation of electrospun nanofibers (ESNFs) as substrate membranes in the fabrication process. As a result, it significantly contributes to the advancement and optimization of TFC FO membrane technology. 

## 3. Effect of Fabrication Methods on the Structural Properties of Supports

**Figure 4:**
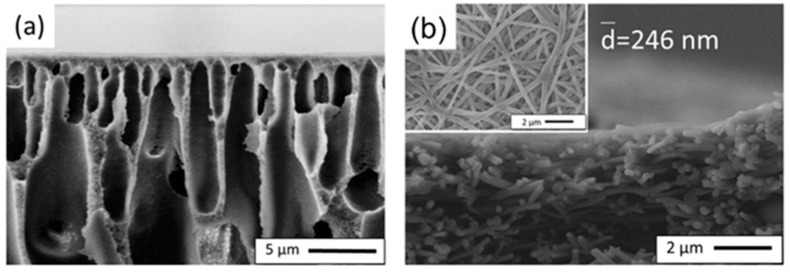
SEM micrographs showing a cross-section of (**a**) asymmetrical and (**b**) nanofibrous PAN substrates. The inset figure shows the surface of hot-pressed PAN nanofiber substrates. Reprinted with permission from Ref. [113]. 2018, Lu et al.

In this review, two methods for the fabrication of supports, namely, the phase inversion method and the ES technique, are presented. Although both ES and phase inversion methods can be used to prepare support layers, differences are observed in the principles, processes, and properties of the resulting membranes. Lu et al. demonstrated the physical configuration of nanofibrous substrates vs traditional phase inversion asymmetrical substrates, where the polymers used were all PAN, as illustrated in Figure 4 [113]. As one of the emerging substrates for TFC membranes, electrospun nanofiber mats (ENMs) exhibit high porosity and low tortuosity of the interconnected pores compared to porous membranes prepared using phase inversion [102]. Mahdavi et al. [112] compared the surface morphologies of PES supports prepared using the phase inversion method and PET nanofibers prepared using the two-axial ES technique. It was observed that the PET nanofibers showed bead-free and large open pore structures and higher porosity (~85%), while, in contrast, the PES support demonstrated a dense structure. The PSf nanofiber support fabricated by Bui et al. [105] using ES technology exhibited notable advantages over the PSf porous support fabricated by Wei et al. [58] using the NIPS method. The former had a thickness of approximately 60.7 μm and a porosity of 81.4%, while the latter had a thickness of 76.1 μm and a porosity of 77%. However, it was noteworthy that a significantly higher contact angle of 132° was measured for the electrospun nanofiber support in contrast to the porous support, which exhibited a lower contact angle of 56°. The membrane structure of the supports and their properties are directly influenced by the different steps and conditions in the preparation process of these two methods [102].

**Figure 5:**
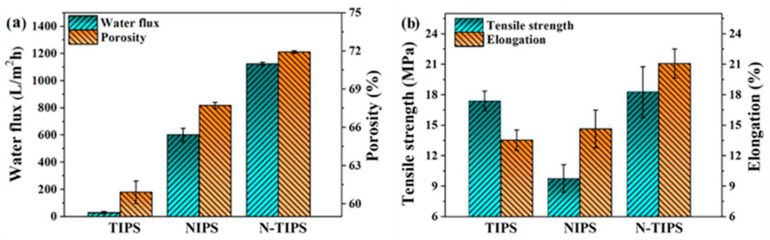
(**a**) Water flux and overall porosity of CTA porous substrates. (**b**) Tensile strength and elongation of CTA porous substrates [76].

The phase inversion method is considered a common method to prepare support layers. Support layers with a desired pore structure and properties can be designed and adjusted with solvent selection, solvent concentration, solution composition, and other factors in the phase separation process. For instance, the PK support structure can be modified during the phase separation process by changing the methanol/water composition of a coagulation bath, as reported by Guan et al. [83]. As the methanol concentration increased, the PK membrane exhibited larger mean pore size, higher porosity water, and permeability. The substrates obtained were also influenced by the method of phase separation induction. CTA porous substrates prepared using TIPS, NIPS, and N-TIPS methods were studied by Han et al. [76]. The smoothest top surface was observed on the CTA_N-TIPS_ substrate, exhibiting an average roughness value of 4.46 nm, which was significantly lower compared to CTA_TIPS_ (17.75 nm) and CTA_NIPS_ (9.24 nm). In addition, the surface pore size of CTA_N-TIPS_ was 11.1 nm, and the highest overall porosity was 67.7%. The largest water flux of 1124.0 L/m^2^ h bar was displayed by the CTA_N-TIPS_ substrate, concurrently demonstrating the best tensile strength and elongation compared to those prepared using NIPS and TIPS processes, as shown in Figure 5. In addition to the incorporation of carbon nanomaterials (oxidized multi-walled carbon nanotube (O-MWCNT) and graphene oxide (GO)) into PES substrate using the phase inversion method by Behdarvand et al. [78], they found that adding GO/MWCNTS to the PES substrate membrane changed the structure of the membrane. Specifically, the addition of abundant oxygen-containing functional groups increased the hydrophilicity of the casting solution, accelerated the rate of phase inversion, and created large finger-like structures that led to increasing the overall porosity [114]. Synthetic hydrophilic rod-like porous nano-hydroxyapatites (PNHAs) were doped into a PSf casting solution to form a support layer using phase inversion in a study by Wang et al. [115]. The results showed that the method not only greatly improved the effective porosity and the connectivity of the channels, but also decreased the thickness of the substrate. Importantly, the mechanical properties of the substrate were enhanced due to the strong interfacial interaction between PNHAs and support.

**Figure 6:**
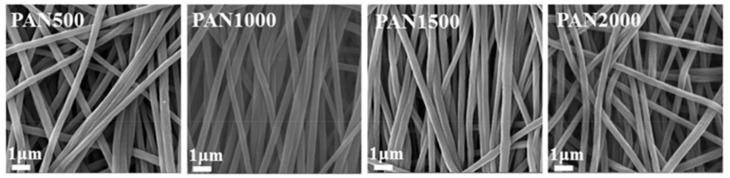
SEM images showing randomly orientated nanofiber substrates (PAN500 and PAN2000) and aligned nanofiber substrates (PAN1000 and PAN1500) obtained at different rotating speeds. The numbers 500, 1000, 1500, and 2000 represent different rotating speeds (rpm). Reprinted with permission from Ref. [103]. 2020, Han et al.

One of the main advantages of the ES technique is that the nanofiber morphology can be controlled using several parameters. The categorization of parameters that can be used to optimize the ES process is divided into three categories: solution parameters, process parameters, and environmental conditions [98]. Bui et al. [105] investigated the surface morphology of PSf in bi-solvent systems of DMF and NMP at various solvent ratios. The results demonstrated a clear relationship between the DMF ratio and the characteristics of the PSf support layers. Increasing the DMF solvent ratios resulted in the formation of smoother nanofiber supports, while the presence of beads and microspheres decreased significantly. Han et al. [103] reported that the degree of nanofiber alignment was determined by the rotating speed of the collector, as shown in Figure 6. Increasing the rotating speed from 500 to 1500 rpm resulted in a more organized alignment of the nanofibers along the rotation direction induced with the rotation of the collector. This can be attributed to the impact of the rotating speed on the trajectory of the continuous ES jet. However, at a higher rotating speed of 2000 rpm, the nanofibers exhibited random orientation due to the excessive speed, which disrupted the attachment of the continuous ES jet and interrupted the alignment of the nanofibers [116]. Furthermore, both temperature [117,118] and humidity [119,120] play respective roles in the ES process. For example, PAN fiber diameters were increased from 150 to 630 nm when the relative humidity increased from 0% to 60%, and mechanical properties in the range of 0% to 40% relative humidity first increased with increasing relative humidity and then exhibited a sharp decrease [121].

## 4. Effect of Structural Properties and Patterns in Supports on the Performance of TFC Membrane

The stable properties of substrate membranes are important for TFC membranes to function in real-life applications [18,122,123]. In the ideal state, the functions of the membrane are unaffected by the performance of the substrate membrane, and the mechanical effect of the substrate merely serves as a platform for the formation of the selective layer. However, in actual situations, the performance of the membrane is influenced by factors such as the hydrophilicity of the substrate membrane [124], the aperture parameters [32,33], and other factors [76,83,98] such as methods used for immersion in water baths [125]. The polymerization, deposition, and adhesion of the active layer, as well as the structural and functional properties of the active layer, are influenced by the support layer. These key properties not only directly affect the performance of TFC membranes in applications [34] but are also important because the membrane will ensure durability during a long period of operation [18]. Therefore, a range of modification techniques are utilized to optimize substrate membranes, aiming to attain an ideal substrate characterized by low bending, high porosity, and hydrophilicity [64,103,126]. This facilitates the development of a tailored membrane that meets specific production requirements, ideally demonstrating exceptional chemical and mechanical stability under elevated pressure conditions while maintaining the desired water separation permeability.

### 4.1. Mechanical Stability

The strength, stiffness, stability, and durability of the overall TFC membranes are significantly influenced by the support layers, which play a crucial role as the structural foundation of the TFC membranes [127,128]. Crucial factors such as the duration of the operating cycle and the efficiency and longevity of the membrane separation process are directly impacted by the support layers [129]. The selection of a support layer should take into account the application environment and requirements to ensure that sufficient mechanical strength is achieved with the TFC membrane during the progress of its use [130,131]. Sustained performance, prevention of premature failure, and maintenance of consistent separation capabilities throughout the operational lifespan are ensured with the use of a substrate membrane with robust mechanical properties. 

When evaluating the mechanical strength of TFC membranes, key metrics such as tensile strength, Young’s modulus, and elongation at the break of supports are considered. Sahebi et al. [77] observed that the membrane surface morphology and hydrophilicity were improved with sulphonation of the PES substrate. Furthermore, the mechanical strength of the membrane was found to be influenced by the degree of sulphonation. Specifically, a significant decrease in tensile strength and Young’s modulus was observed with increasing sulfonation, whereas elongation at the break increased from 17.9% to 43.3%. The reduction in tensile strength caused by sulfonation may be attributed to two factors: reducing the aggregated state and imparting more flexibility to the polymeric material [132,133]. The mechanical properties of the porous substrate can be changed with the introduction of nanofillers, thus enhancing the mechanical stability of TFC membranes [134]. Various concentrations of GO with a superior intrinsic strength were incorporated into the PSf polymer solution to fabricate a highly porous support layer using phase inversion [128]. The highest tensile strength of the PSf/GO nanocomposite support layer (~1.42 MPa) was approximately 78% greater than the support layer without GO (~0.80 MPa). Furthermore, a layered double-hydroxide ((LDH)/GO) hybrid was proven to be a feasible nanofiller for PSf substrates [135]. While the methods used to improve the strength of supports are not limited to those mentioned, the remaining common goal is the effective enhancement of the mechanical stability of TFC membranes. These enhancements ensure the long-term and reliable functionality of TFC membranes for separation, filtration, and other applications.

In general, electrospun nanofiber membranes have a loose structure, which facilitates penetration. However, lower mechanical strength is exhibited by electrospun nanofiber support layers compared to porous support layers. This is caused by the electrostatic repulsion between like charges, thereby maintaining the nanofibers at a certain distance [136]. Hence, in order to overcome the drawback, hot pressing is regarded as an effective strategy for enhancing the mechanical performance of electrospun nanofiber membranes [137,138]. For instance, Wang et al. [139] applied a heat treatment at 240 °C for 1 h after ES, which resulted in a transition from a loose structure to a more compact and integrated structure. Based on the stress–strain curves produced during their study, the stiffness and ductility of the membranes were enhanced with the heat treatment. More recently, Kallem et al. [140] prepared electrospun blended substrate membranes for continuous thermal rolling pretreatment before the IP reaction. It was observed that the thermally treated ESNF substrates consistently exhibited a better tensile strength of 7.5 MPa than the non-thermally treated ESNF substrates. As proposed by Park et al. [141], the mechanical strength of an electrospun PVDF substrate exhibited a remarkable improvement with an approximately 80% increase after heat treatment in comparison to the non-heat-treated PVDF substrate. Similar after-treatment effects have been described in other studies referenced in the literature. In addition, polydopamine (PDA) with good adhesion was used to modify PSf hot-pressed electrospun nanofiber mats (HPENMs) designed by Meng et al. [106]. Noteworthy, the tensile strength (5.7 MPa) and Young’s modulus (215.3 MPa) of HPENMs increase significantly compared to those of virgin ENMs (0.8 MPa and 42.0 MPa, respectively). Similarly, another study prepared a nanofiber substrate by vacuum filtrating an interlayer composed of polydopamine nanoparticles (PDA NPs) onto an electrospun PAN [142]. The T-peel test demonstrated a substantial increase in the adhesion strength between the selective layer and substrate within the TFC FO membrane. Notably, this enhancement was attributed to the effective chemical bonding and entanglement between the polyamides and the PDA NPs, as well as the robust adhesion between the PDA NPs and the nanofibers. These modifications not only bolstered the mechanical strength but also enhanced the stability and water flux of the TFC membranes. For industrialization, it is essential for effective electrospun nanofiber membranes used in industrial production to exhibit robust mechanical stability. 

### 4.2. Polymerization, Deposition, and Adhesion of the Selective (Active) Layer

**Figure 7:**
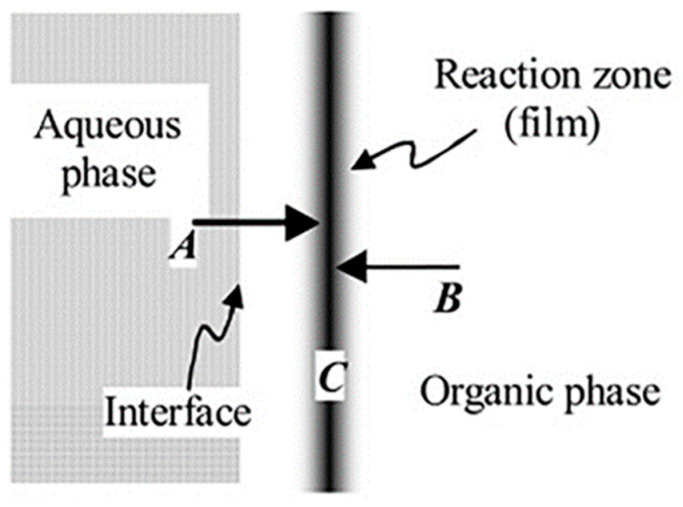
Schematic depiction of IP. Reactant A (initially dissolved in the aqueous phase) and reactant B (dissolved in the organic phase) diffuse toward one another and react to form polymer film C. Reprinted with permission from Ref. [148]. 2005, Viatcheslav.

Traditionally, TFC membranes are prepared using IP technology, and the membrane formation mechanism is shown in Figure 7. Two multifunctional monomers are initially dissolved in different phases (water and an organic solvent), followed by initiating a polymerization reaction on the surface of the support layer [143]. The resulting polymer membrane forms the selective layer of the TFC membrane that is endowed with the desired separation properties [144]. However, with advancements in membrane science and engineering, new strategies for integrating the support layer and selective layer have emerged [145,146,147]. These modern approaches offer enhanced control over membrane properties and performance. Nowadays, various methods, including polymerization, deposition, and adhesion of the selective (active) layer are used to achieve the desired combination of the support layer and the selective layer. 

**Figure 8:**
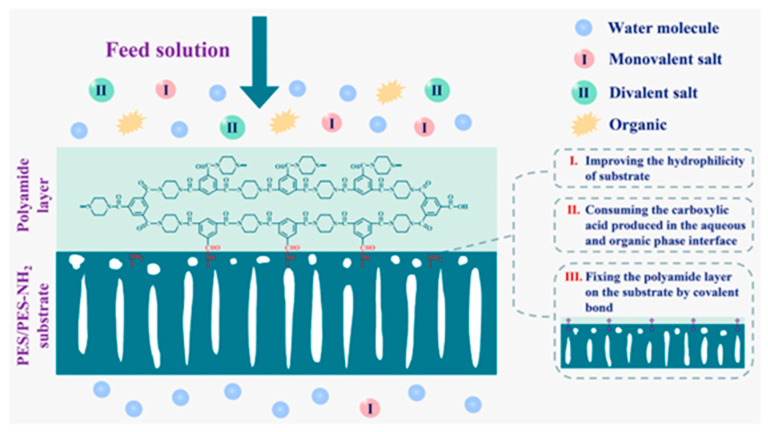
Schematic illustration showing the steps taken to enhance the long-term separation durability of a TFC membrane. Reprinted with permission from Ref. [129]. 2021, Geng et al.

The formation of a selective layer with the IP reaction does not follow uniform growth and generally proceeds three distinct stages [144]. The growth of the selective layer at the support surface is considered a self-limiting, diffusion-controlled process. Misdan et al. [149] found that different substrates for the IP reaction affected the diffusion rate of monomers, which in turn affected the degree of cross-linking in the selective layer and membrane flux. Huang et al. [150] prepared a PA layer on PES electrospun nanofiber substrate using IP for high performance FO. Interestingly, during the 12 h of operation, little change was observed in the J_s_/*J_w_* values (where J_s_ represents the solute flux through the membrane and *J_w_* is the water flux through the membrane), indicating stability of the FO performance for the optimal TFC membrane. Rahimpour et al. [151] formed a rough and dense PA layer on an asymmetric PES support layer using IP. The retention of NaCl by the TFC membrane was 64% and 67% at 5 and 10 bar, respectively, indicating good stability for selectivity. However, when subjected to significant hydraulic shocks, the delicate PA layer may be prone to damage or detachment from the substrate. Nowadays, various methods are available to mitigate delamination, such as coating [141], introduction of an interlayer [152], plasma treatment [31], etc. [106,153], which can enhance the bonding force between the support layer and the PA active layer. For instance, Geng et al. [129] securely anchored a PA layer to a PES/PES-NH_2_ substrate with the introduction of covalent bonding, as shown in Figure 8. When the IP reaction occurred, the –NH_2_ group on the support layer surface served as a H^+^ acceptor, facilitating the consumption of the carboxylic acid generated during the reaction and thereby promoting the progress of the IP reaction [154,155]. Simultaneously, the mass transfer of the amino groups to the organic phase was facilitated, resulting in an increased reaction activity between the amino groups and trimesoyl chloride (TMC). This facilitation contributed to further enhancement in the interfacial force between the selective layer and the support layer. The experimental findings revealed that the TFC membrane exhibited excellent separation performance even during an extended 15-day filtration. In another study, Yao et al. [33] prepared reactable substrates containing amphiphilic copolymer chains and observed that a greater amount of m-phenylenediamine (MPD) aqueous solution was loaded onto the hydrophilic substrate. In addition, due to the polar groups (hydroxyl groups and ester groups) in the amphiphilic copolymer chains, MPD molecules were more easily captured and enriched around the amphiphilic copolymers to form hydrogen bonding interactions. Recently, a long-term stable and high-performance membrane was developed by Park et al. [156] utilizing state-of-the-art dual-layer slot coating (DSC) technology in combination with PDA coating and oxygen plasma treatment. The results of that study demonstrated superior performance of the developed membrane compared to a commercial membrane. Furthermore, Zhang et al. [152] introduced 1,3,5-triformylphloroglucinol p-phenylenediamine (TpPa) covalent organic frameworks (COFs) (one of the Tp series COFs) as an interlayer with nanorods on a PAN nanofibrous substrate. The membrane could withstand a high pressure of 0.9 MPa with slight rejection reduction, and the rejection remained high (>98.5% under 0.5 MPa) after 24 h nanofiltration evaluation. Xiao et al. [157] reported that a tannic acid (TA)-Fe^3+^ interlayer was introduced as a bridge using a chemical bonding connection between the PA layer and the PDA-modified PE support. The water fluxes in the two membranes exhibited a similar trend. However, the TA4-Fe1 membrane exhibited great specific salt flux stability compared to the PDA-8 membrane. 

**Figure 9:**
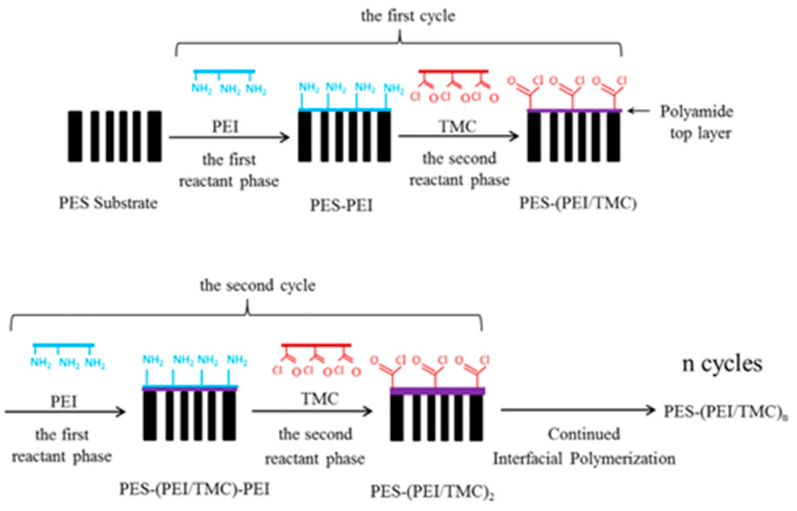
Illustration showing the TFC membrane preparation procedure with multiple cycles of reactant deposition and interfacial polymerization. Reprinted with permission from Ref. [163]. 2014, Wu et al.

Nowadays, in addition to the IP method, an TFC membrane can be prepared using multiple cyclic deposition of reactants, as illustrated in Figure 9. Deposition of a PA active layer on the substrate layer-by-layer (LbL) can form structure similar to IP membranes [158]. Krizak et al. [159] designed TFC membranes by depositing PA layers on levodopa-coated PVDF supports. The membrane exhibited a NaCl rejection of 87.3% and permeability of 52.9 L/m^2^ h bar and performed as a “loose RO” membrane with high permeability and moderate NaCl rejection. A study demonstrated that the performance of an LbL fabricated membrane with an active layer of the appropriate thickness is comparable to that of commercial RO membranes [160]. Choi et al. [161] used an alternative approach termed molecular layer-by-layer (mLbL) for the preparation of TFC membranes. In this method, molecular layers were deposited with the reaction of alternating pendant functional groups, allowing for the creation of highly controlled and tailored membranes. The hydrolyzed PAN support layer was coated with a single bilayer of polyelectrolyte LbL assembly as an intermediate layer, where the PA layer was constructed one monomer layer at a time using cycle alternate cross-linking of the MPD and TMC. A comparison with IP-prepared membranes revealed an NaCl rejection of up to 98.2% and over 2.5 times higher water flux. In addition, cross-linked PA membranes were synthesized with the mLbL progress using PVA as a substrate [158]. Specifically, the TMC solution was deposited on the PVA support layer reacting with pendant alcohol groups. Then, the MPD solution was reacted with the acid chloride groups. Overall, the surface of the mLbL-assembled PA layer was significantly smoother than that produced using IP because of the difference in control methods [160,162]. Their findings demonstrated that the mLbL method allowed for independent control over both intrinsic and extrinsic properties, a feat that could not be accomplished using conventional IP techniques. However, the slow growth rate of the selective layer and the stability of the membrane remain unresolved issues in mLbL.

**Figure 10:**
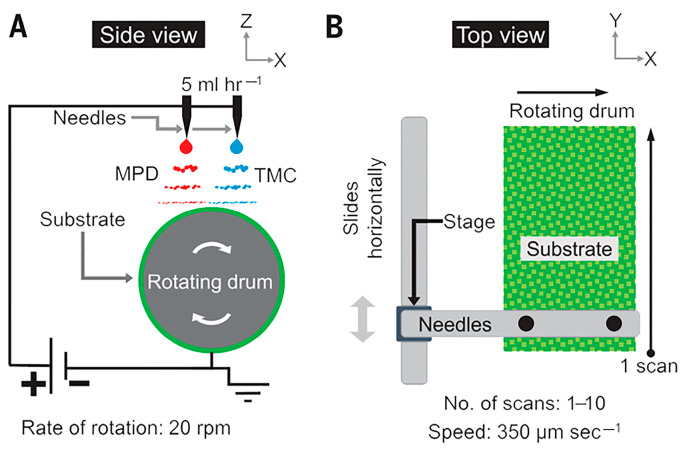
Details of the electrospray process for printing substrate independent polyamide films with thickness control. (**A**) A side view showing a schematic of the electrospray process. (**B**) The top view schematic shows the needles and a stage assembly that can move “horizontally” for uniform coating on a rotated drum. A single sweep across the substrate is denoted as a single scan. Adapted with permission from Ref. [167]. 2018, Chowdhury et al.

In recent years, the manufacturing of TFC-PA membranes using electrospray technology has become possible, and the approach is illustrated in Figure 10. In one study, the PA layer was adhered to the PES support using the sustainable electrospray polymerization (SEP) technique under the action of an electric field and then the membrane performance as evaluated [164]. On the one hand, the membrane exhibited stable performance under various operating conditions and had a roughness lower than that of the IP membrane. On the other hand, the permeability and salt rejection of the SEP membrane was better than those of the IP membrane. Meanwhile, Liu et al. [165] provided a novel strategy to obtain a chitosan (CS)/PES composite substrate using electrospinning of a CS solution onto the surface of PES substrate. The TFC/CS/PES composite membranes were formed after electrospray of 40 min on the CS/PES composite substrate. As a result, the pore size of the substrate was reduced after loading CS nanofibers on the substrate surface, and the adsorption of the amine monomer at the reaction interface was favored over the conventional PES substrate [166]. In addition, the TFC membrane was operated at 4 bar pressure for 100 h and still maintained a high permeability of around 14.2 L/m^2^ h bar and MgCl_2_ rejection of around 10.5 L/m^2^ h bar. However, a longer synthesis time was required for the PA layer due to the slow release of liquid from electrospray, multiple sprayings, and the low rate of speed of the porous substrate. Spray techniques have been widely explored for the preparation of selective layers on porous substrates [147,167,168]. For instance, Wang et al. [169] used ultrafiltration substrates that contained distinct fibrous layers: PET non-woven, electrospun PAN nanofibrous, and ultra-fine cellulose nanofibers (CNs). In the spray process, the aqueous phase was atomized into micron-sized droplets controllably adhering to the substrate to form a selective layer of thinner thickness. The optimized membrane exhibited a permeation flux of 28.6 L/m^2^ h at 0.7 MPa and a NaCl rejection of 96.5%. In another study, Seah et al. [168] dispersed an aqueous solution of piperazine into mist to adhere a PSf substrate, and the TFC membrane achieved a pure water permeability (PWP) of 9.08 L/m^2^ h bar and a Na_2_SO_4_ rejection of 97.2%.

### 4.3. Structural and Functional Properties of the Active Layer

The active layer is considered a key component of the TFC membrane, where the PA layer is mainly developed as the active layer with block-specific molecules and provides paths for water [144,170,171]. The permeation flux test reveals a positive correlation between the TFC membrane roughness and membrane flux, indicating that a TFC membrane with increased roughness exhibits enhanced permeability [34]. Moreover, numerous studies have consistently demonstrated that achieving enhanced permeability in TFC membranes while concurrently enhancing selectivity is a formidable endeavor, notwithstanding extensive exertions and inquiries [172,173]. An essential metric, J_s_/*J_w_* is utilized to evaluate membrane separation selectivity, reflecting the balance between permeability and selectivity [174]. The surface properties and volume characteristics of the substrate membrane significantly impact membrane separation performance [122]. Compared to substrates with larger pores, substrates with a higher surface pore rate or pore density favor the formation of more permeable TFC membranes. This is due to their ability to precisely tune water molecule transport through the membrane’s horizontal transmission path. Moreover, improvement in water permeability can be achieved while maintaining salt resistance. 

**Figure 11:**
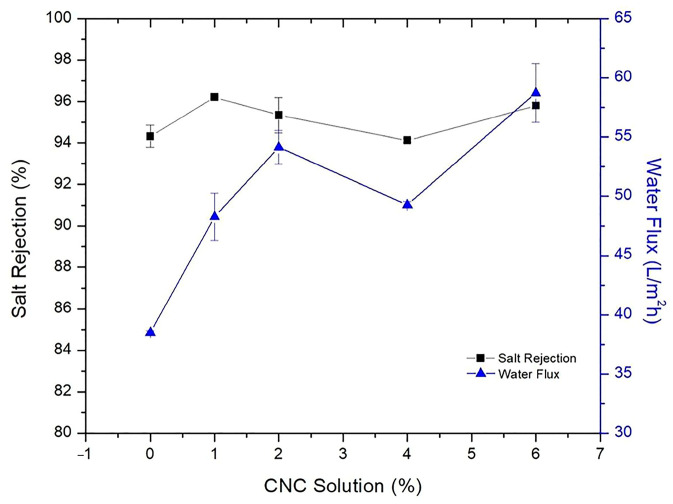
Membrane performance after adding CNC gel to the support layer casting solution [124].

**Figure 12:**
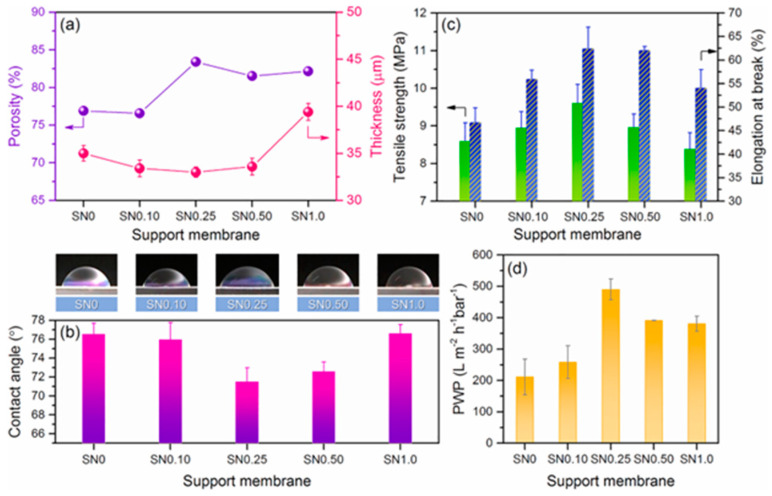
Screening SN membranes for FO support: (**a**) porosity and thickness; (**b**) water contact angle measurements; (**c**) mechanical properties: tensile strength and elongation at break; and (**d**) PWP of SN with different silicene loading using dead−end filtration (Am = 0.9 cm^2^, ΔP = 1.5 bar, membrane compaction for 2 h at 2 bar). The numbers 0, 0.10, 0.25, 0.50, and 1.0 represent the SN loading (wt%). Reprinted with permission from Ref. [178]. 2022, Bendoy et al.

In the modification methods for the support layer, blending is the most widely used modification method to improve substrate membranes due to its simplicity and reliability. In this approach, hydrophilic or amphiphilic additives are introduced into the casting solution to enhance performance. However, achieving good compatibility between the additive and the substrate polymer raw material solution is crucial for exhibiting superior solute selectivity and water permeability [175]. Emadzadeh et al. [64] demonstrated that long finger-like structures formed as the loading of TiO_2_ nanoparticles was increased. The decrease in the contact angle value from 71° to 58° indicated an enhancement in substrate hydrophilicity, which consequently resulted in a greater pure water flux. Specifically, the observed water flux in Emadzadeh et al.’s study was 65% higher than that of the control TFC membrane. In addition, Kadhom et al. [124] added cellulose nano crystal (CNC) gel to a casting solution. The results demonstrated that the water flux increased from 38.5 L/m^2^ h to 54.14 L/m^2^ h with the addition of CNCs from 0% to 2% and maintained the salt rejection around 95%, as shown in Figure 11. Although water flux was highest when 6% CNC gel was added, it disrupted the texture of the sheets unfavorably for long-term operation. In another study, a novel TFC membrane with both positive permeability and dual-layered substrates was prepared by Lim et al. [176] using a co-casting technique. After incorporating GO into the substrate, the membrane was found to exhibit enhanced water flux and ion selectivity. Due to the excellent hydrophilic properties exhibited by the nanofillers, the addition of nanofillers to the support layer was suggested for further enhancement of the overall membrane performance. In a study by Hadadpour et al. [177], the PES substrate was modified by incorporating charcoal-based carbon nanomaterial (charcoal-CNM), resulting in a more rough appearance of the support layer, which played a crucial role in the formation of a highly crosslinked PA selection layer. Furthermore, it was observed that the substrate of the TFC membrane, upon the inclusion of CNM, experienced a significant reduction in the J_s_/*J_w_* value, which markedly reduced from 1.40 to 0.25 g/L. Bendoy et al. [178] designed different silicene nanosheets (SNs) loaded to a PSf substrate. The changes in the support layer porosity, thickness, mechanical strength, hydrophilicity, and PWP with the addition of different SN are depicted in Figure 12. These results demonstrate the positive impact of appropriate loading amounts of SN on the overall performance of the TFC membrane, along with the potential it holds for application in desalination. On the other hand, introducing nanofillers like zeolite nanoparticles [179], non-porous silica [180], zinc oxide nanoparticles [181], and carbon nanotube [182] into the support layer effectively enhanced the hydrophilicity of the membrane surface, thereby resulting in the enhancement of the adsorption and permeability of water molecules through the membrane. Consequently, a significant improvement in the permeability of the membrane was achieved, leading to enhanced efficiency and selectivity in the separation process while maintaining the original purpose. Novel potentials are introduced in the field of membrane separation using this strategy, offering a robust foundation for the achievement of efficient and sustainable separation technologies.

**Figure 13:**
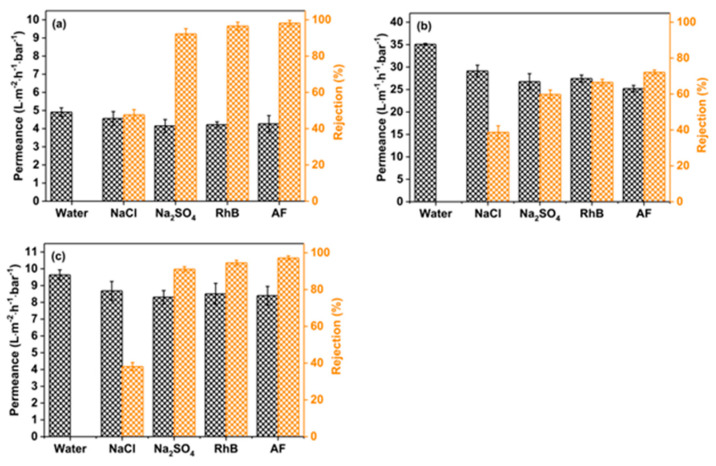
The permeation and separation of TFC (**a**), TFC−ZIF-8 (**b**), and TFC−ZIF8−PSS (**c**) membranes. Reprinted with permission from Ref. [183]. 2021, Zhao et al.

**Figure 14:**
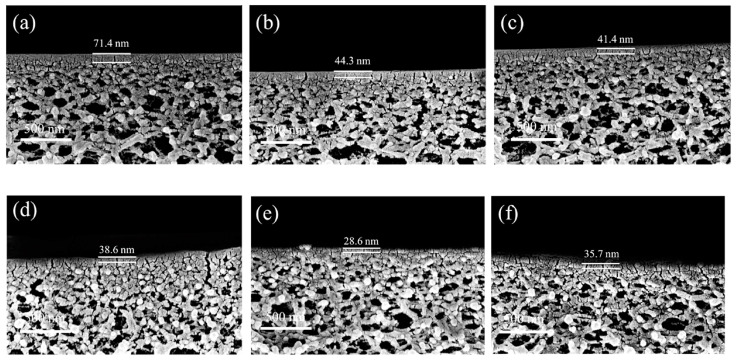
The cross-section morphology of the prepared membranes obtained using SEM. (**a**) NFM-0, (**b**) NFM-2, (**c**) NFM-5, (**d**) NFM-10, (**e**) NFM-15, and (**f**) NFM-20. The numbers 0, 2, 5, and 10 represent the deposition time (min) of PSf substrate membranes soaked into the TTSBI-PEI solution, respectively. Reprinted with permission from Ref. [187]. 2020, Sun et al.

The incorporation of an intermediate layer between the porous support layer and the PA layer has shown promising results for addressing permeability and selectivity, both critical for nanofiltration performance. Notably, Zhao et al. [183] loaded zeolitic imidazolate framework-8 (ZIF-8) onto a PES support layer. As a result, it exhibited a smoother surface with increased negative charge, thereby facilitating the interfacial polymerization process and resulting in a thin, porous, and defect-free PA layer. The synergistic effect between ZIF-8 and poly(sodium 4-styrenesulfonate) (PSS) effectively improved the performance of the nanofiltration membrane, as shown in Figure 13. The diffusion rate of the aqueous phase into the organic phase direction can be controlled with the introduction of an organic intermediate layer, which in turn changes the morphology or thickness of the PA membrane [184,185,186,187]. As presented in Figure 14, the introduction of a (5,5′,6,6′-tetrahydroxy-3,3,3′,3′-tetramethyl-1,1′-spirobisindane) TTSBI-PEI intermediate layer onto a PSf substrate membrane resulted in a remarkably thin active layer with only a 28.6 nm thickness for the nanofiltration membrane (NFM)-15 membrane. Moreover, the design of the nanosheets interlayer has also proven to be an effective method. A study by Lu et al. [188] presented the design of 2D layered double-hydroxide (LDH) nanosheets for modifying the PSf/sPSf support layer. Similarly, Wu et al. [189] used the scalable brush-coating technique for MXene (Ti_3_C_2_T_x_) onto nylon substrates to fabricate FO membranes. The results exhibited improved permeability and salt rejection properties, which shed new light on the feasible application of FO membranes. However, the practical applications of this approach may encounter challenges due to the weak interfacial interactions between the intermediate layer and the porous support.

**Figure 15:**
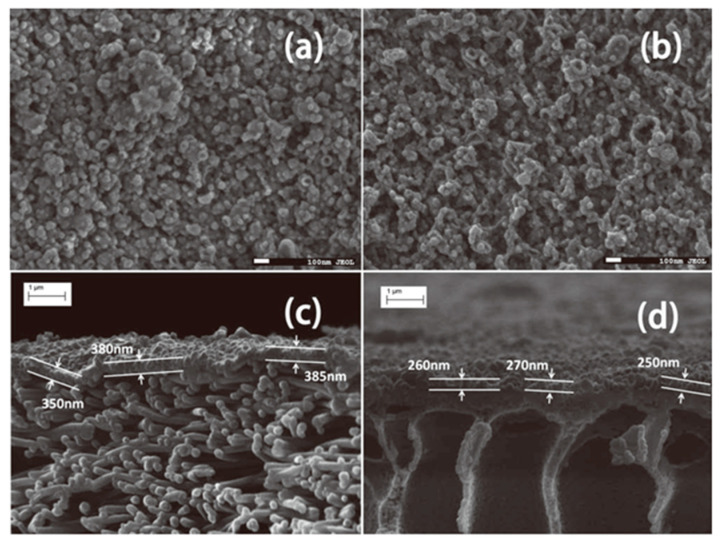
(**a**,**b**) NC-FO and PI-FO membrane surfaces showing typical ridge-and-valley morphology of interfacially polymerized PA membranes. (**c**) Cross-section of the NC-FO membrane suggesting that the PA active layer is about 370 nm ± 20 nm thick. (**d**) Cross-section of the PI-FO membrane suggesting that the PA active layer is about 260 nm ± 10 nm thick. Reprinted with permission from Ref. [102]. 2011, Song et al.

Research on enhancing membrane properties using various modifications of phase inversion porous substrates has been extensively conducted, but it generally requires additional porogen agents to reduce the formation of closed pores. Interestingly, electrospun substrate membranes exhibit tremendous promise in the realm of membrane filtration technology, owing to their high porosity, submicron pore diameter, open pore structure, and so on [190]. Song et al. [102] indicated that the water permeability of an electrospun nanocomposite FO (NC-FO) membrane was approximately 36% higher compared to a phase inversion FO (PI-FO) membrane, which was due to the highly porous nanofiber support layer promoted by the formation of thinner PA active layers, as illustrated in Figure 15. In addition, Tian et al. [109] discovered that TFC membranes fabricated from nanofiber substrate membranes with smaller pore sizes had a better selectivity of 0.46 g/L. Specifically, PA layers with better selectivity and denser layers can be fabricated more easily on substrates with smaller pores and higher roughness [191].

**Figure 16:**
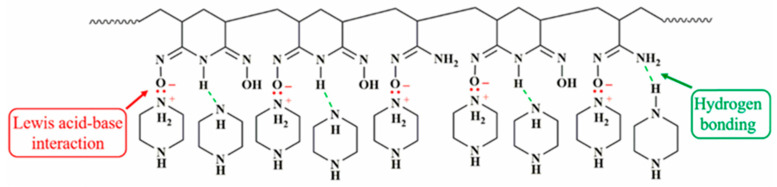
Possible interaction mechanism between PAO and PIP. Reprinted with permission from Ref. [6]. 2022, Liu et al.

**Figure 17:**
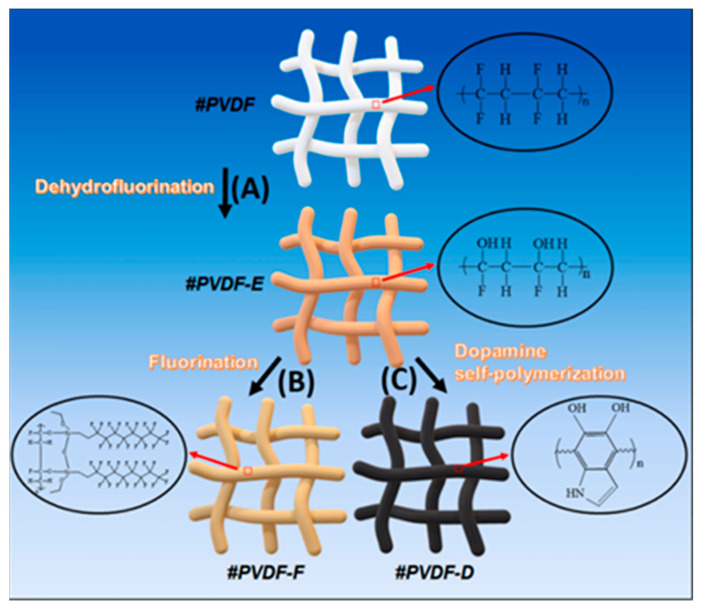
(**A**) Dehydrofluorination of the hydrophobic substrate #PVDF with an alkaline solution to develop the hydrophilic substrate #PVDF-E. (**B**) Fluorination of #PVDF-E with fluorodecyltriethoxysilane (FAS) to obtain the superhydrophobic substrate #PVDF-F. (**C**) Dopamine self-polymerization on #PVDF-E to obtain the superhydrophilic #PVDF-D. The #PVDF, #PVDF-D, #PVDF-E, and #PVDF-F represent nanofibrous substrate, superhydrophilic substrate, hydroxyl-modified substrate, and superhydrophobic substrate, respectively. Reprinted with permission from Ref. [194]. 2022, Huang et al.

Notable improvements in permeability and water flux are observed in emerging ESNF substrate membranes. For example, Al-Furaiji et al. [190] prepared a TFC membrane for the FO process using a PAN electrospun nanofiber support layer. They found a stable water flux of about 16 L/m^2^ h during 20 h of operation and an average salt flux average value of about 4 g/m^2^ h. Additionally, Meng et al. [106] used ESNF as a substrate membrane and found that the introduction of PDA increased the water flux, but the salt retention rate remained stable at 0.064 g/L. Remarkably, Shibuya et al. [111] first report a composite nanofiber substrate fabricated using the coaxial ES technique, and the authors compared the multifaceted TFC membrane performance of PVDF-TFC, CA-TFC, blended-TFC, and composite-TFC. The results showed that composite-TFC exhibited a water flux of 31.20 L/m^2^ h bar and a specific reverse salt flux of 0.03 g/L, which was by far the lowest value reported in the literature while maintaining the advantages of PVDF with better mechanical strength and hydrophilic CA. The introduction of an interlayer into a TFC membrane has been proven to be an effective strategy to tailor the PA active layer [192]. Recently, Liu et al. [6] introduced poly (amidoxime) (PAO) as organic interlayer. The interaction mechanism between PAO and piperazine (PIP)is illustrated in Figure 16. As a result, the novel membrane exhibited a permeance up to 25.2 L/m^2^ h bar with a rejection above 99% for Na_2_SO_4_. Similarly, a hydrophobic PVDF nanofiber substrate was modified using a PDA-GO interlayer [193]. One noteworthy observation was that the PDA-GO interlayer was only modified on the substrate surface rather than being applied to the total substrate. The control TFC had a J_s_/*J_w_* of 0.18 g/L, while the optimal TFC had a J_s_/*J_w_* of 0.04 g/L. Moreover, four nanofibrous substrates with different surface wetting properties were developed by Huang et al. [194]. Their work aimed to fill a research gap regarding the influence of nanofibrous substrate wettability on TFC membranes. The preparation process for the nanofiber substrate with different surface wettability on TFC membrane is shown in Figure 17. As a result, satisfactory J_s_/*J_w_* values lower than 0.2 g/L were exhibited by all the prepared membranes. 

### 4.4. Mitigating Fouling

The issue of membrane contamination remains an eternal topic, with pollution of a membrane potentially being caused by the hydrophobic nature of the substrate membrane. Different types of fouling can be observed in water treatment, including but not limited to heavy metals, organic compounds, dyes, solvents, biological waste, dust, gravel, germs, bacteria, and salts [18]. Pollutants have the capability of being adsorbed onto the membrane surface or the pore walls, thereby obstructing the membrane orifices and resulting in membrane pollution [195]. This, in turn, significantly diminishes the permeation and separation performance of membranes [196]. However, it is crucial to note that frequent cleaning of membranes fails to conform to the principles of economic efficiency, as it also shortens a membrane’s service life when harsh cleaning agents are used [197]. Additionally, certain pollutants can react with the active layer, causing defects in its structure and resulting in the loss of TFC membrane performance. Consequently, enhancing a membrane’s resistance to contamination is of paramount importance in extending its operational lifetime and minimizing economic expenditures [198].

Currently, more attention is still focused on surface modification of TFC membranes, with relatively few studies investigating the improvement of anti-fouling performance using modified support layers. Moreover, TFC membranes prepared using phase inversion methods are relatively more studied for anti-fouling than TFC membranes prepared with electrospun nanofibers as support layers. This is primarily due to the structure of electrospun nanofibers, which has predominantly been studied for its impact on the permeability of TFC membranes [199]. Hydrophilicity and surface roughness are the effective parameters for improving anti-pollution [200]. The creation of hydrogen bonds between the membrane surface and water molecules, as well as the formation of a hydration layer on the membrane surface, are facilitated by the hydrophilicity of the membrane [201]. The interactions between the membrane surface and pollutants are reduced by this hydration layer, which decreases membrane fouling. Numerous investigations have highlighted the effectiveness of hydrophilically modified support layers for mitigating membrane fouling in hydrophobic membranes [202]. A TFC membrane with a low fouling tendency was achieved with the incorporation of highly hydrophilic phosphorylated titanium dioxide (p-TiO_2_) into the PVDF support layer [203]. After subjecting the TFC membranes to a dynamic fouling test with 500 ppm humic acid (HA) solution for 20 h, the results showed that the water flux of the TFC membrane first decreased significantly and then decreased insignificantly. In addition, optimized TFC membranes were applied to model heavy metal and antibiotic wastewater treatment using hydrophilic treatment and hot-pressed modification of electrospun PSf nanofibers [106]. It was observed that the optimized TFC membranes showed a good anti-fouling property for metal ions and antibiotics, with high rejections of 97.9–100%. Behdarvand et al. [78] synthesized a novel thin-film nanocomposite (TFN) membrane with the integration of carbon nanomaterials (oxidized multi-walled carbon nanotube (O-MWCNT) and GO into a PES substrate using the phase inversion method. The PES substrate surface was coated with polyvinyl alcohol (PVA) and subsequently cross-linked with glutaraldehyde (GA). The incorporation of carbon nanomaterials into the PES substrate resulted in a more negative charge on the membrane surface. This increased the electrostatic repulsive force between the membrane surface and foulants, leading to a lower contact and attachment rate. 

The flux recovery rate (*FRR*) is used as an index to evaluate the anti-fouling property of a TFC membrane, which is calculated with the following expression:

(1) $$FRR_{n}=\left(\frac{J_{wn}}{J_{w0}}\right)\times 100\%$$

where the *J_w_*_0_ and *J_wn_* are the initial and the nth cycle (*n* = 1, 2) of pure water flux, respectively.

Furthermore, other methods have also been proven to mitigate fouling. Fang et al. [204] coated zirconium-based metal-organic frameworks (MOFs) onto a substrate and then analyzed the anti-fouling of the TFC membrane. The *FRR* values of the TFC membrane for sodium alginate (SA) and HA reached 95.0% and 92.2%, respectively. Consequently, the TFC membrane exhibited exceptional anti-fouling properties, effectively combating the erosion caused by organic and biological foulants. These methods can improve membrane hydrophilicity and limit the adsorption between the contaminants and the membrane surface to decrease membrane-fouling propensity. Interestingly, a tiered dual-layer nanofibrous substrate was composed of a coarse fiber layer (100–300 nm), and a fine fiber interlayer (40–60 nm) was studied [205]. Meanwhile, the anti-fouling of the TFC membrane draw solution (DS) and feed solution (FS) was evaluated using SA and bovine serum albumin (BSA). After physical cleaning, the *FRR* values of the TFC membrane for SA and BSA reached 78.1% and 88.5%, respectively. The development of a contamination layer on the membrane surface in NaCl-containing solutions can be greatly influenced by more vital intermolecular SA, thereby causing increased membrane surface contamination.

## 5. Conclusions and Future Prospective

Overall, research on support layers has paved the way for the development of more robust and efficient TFC membranes. In the design and development of TFC membranes, the selection of an appropriate support layer is crucial to maximize membrane performance. The support layer obtained using the phase inversion method possesses a uniform and continuous structure, ensuring adequate mechanical strength and stability. On the other hand, electrospun nanofiber support layers are known for their high specific surface area and open pore structure with low resistance and high flux, providing superior permeability and separation performance. Various methods have been used to modify the surface properties and intrinsic properties of the support layer, including blending, introduction of intermediate layer, coating, introduction of functional groups, etc., to improve the overall performance of TFC membranes. Considering the different types of support layers, selection of the most suitable one for a particular application depends on factors such as the desired separation effect, flux requirements, membrane stability, and material cost.

As far as conventional substrate membranes are concerned, their advantage lies in their suitability for large-volume preparation, making them favored for industrial production and application. Moreover, superior mechanical stability, enhanced reusability, and suitability for long-term operations are exhibited by TFC membranes prepared with traditional substrate membranes. However, additional pore-forming methods are typically used to impart the required pore properties to the substrate membrane, which adds a post-processing step for the removal the porogen agent. Furthermore, the flux in a TFC membrane is limited due to the poor porosity and closed pores of the support layer, thus higher pressure may be required during operation. The introduction of an intermediate layer is considered to improve the hydrophilicity of the substrate membrane to some extent, thereby increasing the membrane flux. On the other hand, it also increases the possibility of TFC membrane layer stripping and difficulties in membrane cleaning. Therefore, the search for substrates with better hydrophilicity and chemical stability, the improvement of pore-forming methods, and the incorporation of nanomaterials to prepare oriented pores are considered the main approaches to overcome the drawbacks of traditional substrate membranes. Meanwhile, the complexity of their preparation methods, their compatibility with the substrate, and the economic benefits they bring need to be taken into consideration when introducing new materials such as MXene.

The preparation method for nanofiber substrate membranes is relatively simple as it does not require additional porogen agents or closed pores. Additionally, its controllable thickness allows for the facile fabrication of thin active layers, consequently leading to higher membrane flux. However, its mechanical strength is relatively low, and additional processing is required to enhance its mechanical properties. Despite the feasibility of large-scale production, the substantial solvent volatilization can cause environmental concerns. Therefore, the primary challenge in this field is to seek green chemistry (environmentally friendly) methods for the large-scale fabrication of nanofiber membranes. In addition, the loss of membrane mechanical properties at higher pressures can be caused by the potential breakage of nanofibers, which in turn affects the overall performance of the membrane. Although research in this field is relatively lacking, it is a crucial aspect that must be considered for the long-term operation of TFC membranes. To address this issue, comprehensive investigations can be conducted on controlling fiber material, diameter, substrate membrane thickness, and post-processing techniques. Meanwhile, the coaxial electrospinning method (such as introducing PVDF, carbon fibers, etc., as core material) can be considered to prepare composite nanofiber substrate to improve mechanical strength.

Therefore, both traditional substrate membranes and nanofiber membranes have entered the period of precise control of the structure to address their respective primary issues. In future research, one of the avenues to be explored is to combine these two approaches to investigate the feasibility of using nanofibers to construct a substrate membrane skeleton and then pouring a casting solution to prepare a composite-based membrane, which would greatly improve the mechanical properties and long-term operational performance of the membrane. In addition, the use of nanofibers as a porogen agent enables the substrate membrane to have long and intact water channels, thereby overcoming the problem of more closed pores commonly found in traditional substrate membranes and increasing water flux, reducing operating pressure, and decreasing the long-term operating costs of the membrane system.

## Figures and Tables

**Table 1:** Fabrication process and parameters for substrate membrane preparation.

Substrate	Method	Solvent	Polymer Additives	Coagulation Bath	Coagulation Temperature (°C)	Year	Reference
PSf	NIPS	N-methyl-2-pyrroli-done (NMP)	polyvinyl pyrrolidone (PVP)	water	23	2011	[58]
PSf	NIPS	NMP	PVP/lithium chloride (LiCl)	water	23	2011	[59]
PSf	NIPS	NMP	diethylene glycol (DEG)	water	25	2012	[60]
PSf	NIPS	NMP/dimethylfor-mamide (DMF)	-	water	24.5	2021	[61]
PSf	NIPS	NMP	-	water	22	2014	[62]
PSf	NIPS	NMP	polyethylene glycol (PEG)-400	water	25	2019	[63]
PSf	NIPS	NMP	PVP/titanium dioxide (TiO_2_)	water	25	2014	[64]
PSf	NIPS	DMF	-	water	25	2018	[65]
PSf	NIPS	DMF	PVP	water	15, 20, 25, 30	2019	[36]
PSf	NIPS	DMF	-	0.1% (*w*/*v*) sodium lauryl sulfate in H_2_O/DMF (H_2_O:DMF = 96:4 *v*/*v*)	24–25	2014	[29]
PSf	NIPS	N,N-dimethylaceta-mide (DMAc)	PEG/PVP	water	60	2022	[66]
PSf/sPSf	NIPS	NMP	-	water	25	2018	[67]
PVDF	NIPS	NMP	perfluorosulfonic acid	water	25	2017	[51]
PVDF	NIPS	DMAc	-	water	25	2017, 2016	[68]
PVDF	NIPS	DMAc	silicon dioxide (SiO_2_)/PVP	water	25	2019	[69]
PVDF	VIPS	NMP/DMAc/DMF	-	water	25	2010	[70]
PVDF	VIPS	DMAc	PVP/LiCl	water	25	2021	[71]
PVDF	TIPS	Triethyl phosphate (TEP)	PEG-400	water	10	2017	[42]
PAN	NIPS	DMF	-	water	25	2015	[72]
PAN	NIPS	NMP	-	water	20	2013	[73]
PAN	NIPS	NMP/DMF	-	water	25	2016	[74]
PAN	NIPS	NMP	-	water	25	2019	[75]
cellulose triacetate (CTA)	NIPS	dimethylsulfone (DMSO_2_)	PEG-400	water	95	2022	[76]
CTA	TIPS	DMSO_2_	PEG-400	glycerin	50	2022	[76]
CTA	NTIPS	DMSO_2_	PEG-400	water	50	2022	[76]
PES/Sulphonatedpolyethersulfone(SPES)	NIPS	NMP	-	water	25	2016	[77]
PES	NIPS	DMAc	-	water	25	2021	[78]
PES	NIPS	NMP	PEG-2000	water	25	2022	[79]
PES	NIPS	NMP	PVP	water	25	2021	[49]
PES	NIPS	NMP/DMF	-	water	24.5	2021	[61]
PES	NTPS	DMAc	DEG/PEG	water	25	2017	[80]
CA	NIPS	acetone (Ac)/DMAc	PVP/PEG	water	25	2017	[81]
polyvinyl- chloride (PVC)	NIPS	DMAc	PEG-400/LiCl	water	25	2018	[33]
PVC	NIPS	NMP	PVP	water	25	2018	[82]
polyketone (PK)	NIPS	resorcinol/water	-	methanol/water	25	2021, 2021	[83,84]
polyetherimide (PEI)	NIPS	NMP/DMF	-	water	24.5	2021	[61]
PI	NIPS	dimethylsulfoxide (DMSO)	-	water	21	2013,2012	[10,85]
PI	NIPS	DMF	-	water	21	2013	[10]
PI	NIPS	NMP	-	water or 0.1 M NaOH solution	25	2022	[86]
PI	NIPS	DMSO/DMF	-	water	22	2014	[62]
PEEK	NIPS	methane sulfonic acid (MSA) and sulfuric acid (H_2_SO_4_)	-	water	21	2013	[10]
PES/sulfonated polyetheretherketone (sPEEK)	NIPS	NMP	-	water	25	2017	[87]
PC	NIPS	NMP	PVP	water	25	2021	[49]
poly(hydroxyamide) (PHA)	NIPS	NMP	-	water	30	2018	[88]

**Table 2:** Preparation of nanofiber substrate membranes.

Substrate	Solvent	Voltage (kV)	Distance (cm)	Flow Rate (mL/h)	Temperature (°C)	Humidity (%)	Fiber Diameter (nm)	Reference
PES	NMP/DMF	25	15	0.6	–	–	–	[102]
PAN	DMF	20	15	0.8	–	–	475 ± 92	[103]
PAN/CA	DMF	28.5	16	1.0	25	50	–	[104]
PSf	DMF/NMP	27.5	16	1.2, 0.9, 0.6	25	10	250	[105]
PSf	DMF/NMP	25	12	1.0	25	25–30	–	[106]
PVDF/PAN	DMAc	18–21	16	0.8–1.0	25	–	745 ± 281	[107]
polyimide (PI)	DMF/NMP	60	18	–	21–23	30 ± 2	–	[108]
PVDF	DMF	27	12	0.18	25	70	280 ± 80	[109]
PVDF	DMF	18	25	1.5	25	–	302	[110]
PVDF	DMAc/acetone	21–24	18	2.0	22–25	40–60	1260.6 ± 308.9	[111]
CA	DMAc/acetone	21–24	18	2.0	22–25	40–60	259.2 ± 114.6	[111]
CA/PVDF	DMAc/acetone	21–24	18	2.0	22–25	40–60	788.6 ± 326.3	[111]
polyethylene terephthalate (PET)	trifluoroacetic acid (TFA)/dichloromethane (DCM)	25	12	1.2	25	–	105 ± 10	[112]

## Data Availability

Not applicable.
